# Auranofin and Pharmacologic Ascorbate as Radiomodulators in the Treatment of Pancreatic Cancer

**DOI:** 10.3390/antiox11050971

**Published:** 2022-05-14

**Authors:** Garett J. Steers, Gloria Y. Chen, Brianne R. O’Leary, Juan Du, Hannah Van Beek, Joseph J. Cullen

**Affiliations:** 1 Free Radical and Radiation Biology Program, Department of Radiation Oncology, The University of Iowa Carver College of Medicine, Iowa City, IA 52242, USA; garett-steers@uiowa.edu (G.J.S.); gloria-chen@uiowa.edu (G.Y.C.); brianne-oleary@uiowa.edu (B.R.O.); juan-du@uiowa.edu (J.D.); vanbeekhm@outlook.com (H.V.B.); 2 Department of Surgery, The University of Iowa Carver College of Medicine, Iowa City, IA 52242, USA

**Keywords:** pharmacologic ascorbate, vitamin C, pancreatic cancer, Auranofin, thioredoxin, thioredoxin reductase, peroxiredoxin

## Abstract

Pancreatic cancer accounts for nearly one fourth of all new cancers worldwide. Little progress in the development of novel or adjuvant therapies has been made over the past few decades and new approaches to the treatment of pancreatic cancer are desperately needed. Pharmacologic ascorbate (P-AscH^−^, high-dose, intravenous vitamin C) is being investigated in clinical trials as an adjunct to standard-of-care chemoradiation treatments. In vitro, P-AscH^−^ has been shown to sensitize cancer cells to ionizing radiation in a manner that is dependent on the generation of H_2_O_2_ while simultaneously protecting normal tissue from radiation damage. There is renewed interest in Auranofin (Au), an FDA-approved medication utilized in the treatment of rheumatoid arthritis, as an anti-cancer agent. Au inhibits the thioredoxin antioxidant system, thus increasing the overall peroxide burden on cancer cells. In support of current literature demonstrating Au’s effectiveness in breast, colon, lung, and ovarian cancer, we offer additional data that demonstrate the effectiveness of Au alone and in combination with P-AscH^−^ and ionizing radiation in pancreatic cancer treatment. Combining P-AscH^−^ and Au in the treatment of pancreatic cancer may confer multiple mechanisms to increase H_2_O_2_-dependent toxicity amongst cancer cells and provide a promising translatable avenue by which to enhance radiation effectiveness and improve patient outcomes.

## 1. Pancreatic Cancer

Pancreatic ductal adenocarcinoma is one of the most common cancers worldwide and has remained a leading cause of cancer deaths. In the United States, the estimated incidence is 13.2 cases per 100,000 people, with a mortality rate of 11.1 per 100,000 people, making it the third leading cause of cancer deaths after lung and colorectal cancer [1,2]. Despite a small upward trend in 5-year survival rate since 1975, survival remains the lowest among all cancer types globally, with a current 5-year survival rate of 10.8% for all stages [2,3]. Similarly, the mortality rate for pancreatic cancer has not changed in nearly 50 years; by comparison, colorectal cancer, the second most common cause of cancer deaths, has seen a drop in mortality rate of nearly 60% over the same period [1]. This poor prognosis is attributed to the delayed diagnosis and poor response to therapy. As a result, incidence and mortality rates have increased throughout all regions of the world and are predicted to continue to rise [3].

Treatment of pancreatic cancer is complex and often requires a multi-disciplinary approach given its high mortality and morbidity. Primary factors affecting prognosis and survival are resectability and metastasis. The resectability of a tumor is based on its relation to nearby structures as well as the degree of lymphatic spread. Tumors that invade or encase nearby vasculature, distort vascular anatomy, or invade other nearby organs such as the stomach are considered borderline resectable or locally advanced, indicating a need for neoadjuvant chemoradiotherapy prior to reevaluation for resection [4]. In a similar fashion, the degree of disease in the regional lymph nodes also guides the decision for resection. Distant lymph nodes outside of the field of resection are indicative of more distant, metastatic disease, where upfront resection is avoided. Only approximately 13% of patients are considered resectable at the time of their diagnosis; thus, neoadjuvant chemoradiotherapy is critical in managing more extensive disease with the goal of converting locally advanced, unresectable tumors to resectable [1,5]. Rates of survival for resected patients have increased to as high as 17.4% in 2011 from 1.5% in 1975, while the survival rate of non-resected patients has expectedly remained unchanged during this time, less than 1% [3,6].

For both resectable and unresectable tumors, chemotherapy is a vital component of pancreatic cancer treatment. Patients who undergo initial surgical resection without evidence of recurrence or metastatic disease postoperatively will still require systemic adjuvant treatment [4]. In several multi-center randomized controlled trials, FOLFIRNOX (a combination treatment of fluorouracil, leucovorin, oxaliplatin, and irinotecan) improved overall survival and progression-free survival compared to gemcitabine alone and is now considered standard of care for both neoadjuvant and adjuvant therapy [7,8]. Additional chemotherapy regimens such as gemcitabine plus nab-paclitaxel also offer survival benefit [9]. For locally advanced disease requiring neoadjuvant therapy prior to the consideration of resection, several adjuncts to chemotherapy are available, including radiation therapy, thermal and non-thermal ablation, and intra-arterial chemotherapy. These treatments have varying amounts of success but have been seen to improve overall prognosis [10].

Radiation Therapy as Adjunct Treatment for Pancreatic Cancer

Radiation is primarily known to cause direct DNA damage [11,12,13,14]. It also generates reactive oxygen species, which cause further damage at the DNA level with single- and double-stranded DNA breaks, as well as damage to proteins and lipids [15]. Radiotherapy is often used in conjunction with chemotherapy to increase radiation effectiveness. In pancreatic cancer, fluorouracil (5-FU) was classically utilized, while gemcitabine, capecitabine, and oral 5-FU derivatives such as Xeloda are commonly used today [4]. Neoadjuvant treatment with radiation aims to improve the likelihood of margin-negative resection or downsizing of tumors, although there is no standard practice [16]. In locally advanced and unresectable disease, radiation may assist in local control or prevent local progression or recurrence, despite no evidence of improved overall survival in the limited studies available. A phase III randomized controlled trial, LAP07, investigated locally advanced pancreatic cancer receiving chemoradiotherapy compared to chemotherapy alone. Although there was no survival benefit, there were some improvements in local tumor progression and time to re-initiation of therapy [10,16,17]. As adjuvant therapy, chemoradiation has varying results, with no significant benefit in overall survival, but it has shown some potential in certain patients, particularly those with positive margins [18]. Radiation therapy is also used as an aid in relieving pain, bleeding, or obstructive symptoms in palliative or metastatic disease. Advances such as intensity-modulated RT (IMRT) and stereotactic-body RT (SBRT) have enabled more dose escalation and reduced volumes to improve clinical outcomes [10]. These may be better tolerated by patients and investigations are underway to further assess their role in pancreatic cancer treatment [19,20,21].

The efficacy of radiotherapy has been restricted due to the toxic effects on patients’ quality of life. Due to the location of the pancreas, many nearby organs, such as the small intestine and stomach, are directly within the radiation field. The standard dose is then limited to 50–54 Gy to maintain local control of the cancer without significant toxicity and side effects [10]. Common significant side effects include hematologic toxicities, GI symptoms such as nausea, diarrhea, and abdominal pain, and biliary obstruction. These side effects lead to decreased tolerance of radiation, poorer quality of life for patients, and a limited effect of the radiation itself [22]. Despite increasing the intensity of treatment by altering radiation fractionation or with different concurrent chemotherapies, the majority of patients still experience local failure and succumb to their disease [23]. Thus, there are a significant number of pancreatic cancer patients who would benefit from improvements in the efficacy of standard-of-care chemoradiation therapy.

## 2. Pharmacologic Ascorbate

Vitamin C (ascorbic acid, ascorbate) at normal physiologic doses is typically considered a donor antioxidant. Ascorbic acid (AscH_2_) readily oxidizes to ascorbate (AscH^−^), which then undergoes two one-electron oxidations to form the ascorbate radical (Asc^−^) [24]. The ascorbate radical is relatively unreactive and can combine with hydrogen ions to reform ascorbate, making ascorbate an effective antioxidant at physiologic levels [24]. However, at supraphysiologic levels, ascorbate exerts pro-oxidant effects. Intestinal absorption of ascorbate is tightly regulated by sodium-dependent vitamin C transporters in enterocytes, with average serum levels maintained around 40–80 µM [25,26,27,28,29]. Thus, supraphysiologic levels cannot be obtained by oral supplementation.

Intravenous administration of ascorbate circumvents this limitation. Pharmacologic ascorbate (P-AscH^−^, high-dose intravenously administered ascorbate) produces much higher serum ascorbate levels, up to 20 mM [30]. At these supraphysiologic levels, ascorbate acts as a pro-oxidant, donating electrons to form high levels of extracellular hydrogen peroxide (H_2_O_2_) that readily crosses cell membranes [24,25,31]. Once H_2_O_2_ is intracellular, it reacts with redox-active metals to form the hydroxyl radical (HO^∙^), a highly toxic radical that causes oxidative damage to proteins, lipids, and DNA [24,31,32,33]. Due to this pathway, P-AscH^−^ has garnered significant interest as a potential cancer therapy.

### 2.1. Pharmacologic Ascorbate Use in Cancer

The use of vitamin C for the treatment of cancer dates back to the 1970s, when Cameron et al. suggested that high-dose ascorbate could provide a survival benefit when used to treat a variety of cancers, including stomach, colon, rectal, breast, bladder, and pancreatic cancer [34,35,36]. In these studies, patients considered to have terminal disease were given intravenous ascorbic acid (typically 10 g/day) in addition to the standard of care at that time. They observed significantly increased survival times in the P-AscH^−^ treated patients compared to the controls, results which would be demonstrated again in subsequent studies [35,36]. However, in the late 1970s and mid-1980s, two double-blinded, randomized controlled trials studying the use of ascorbate in the treatment of several solid organ tumors demonstrated no effect on patient survival [37,38]. These studies slowed the adoption of P-AscH^−^ in cancer research and treatment. However, these studies differed significantly from those performed by Cameron in that patients received only oral ascorbate as opposed to high-dose ascorbate administered intravenously. As described above, oral supplementation of ascorbate fails to elevate serum levels above the normal physiologic threshold [25,27,28,29]. These findings in the late 1990s and early 2000s ignited a resurgence in interest around P-AscH^−^ in the treatment of cancer.

Pharmacologic ascorbate has shown promise in the treatment of a variety of cancers over the last decade. In vitro and in vivo studies in pancreatic, breast, colorectal, ovarian, glioblastoma, and lung cancer have demonstrated that P-AscH^−^ decreases cell viability, decreases tumor volume, and improves survival alone or in combination with other standard-of-care chemotherapy regimens [33,39,40,41,42,43,44,45]. The selectivity of ascorbate-induced cytotoxicity between normal cells and malignant cells may be due to lower levels of peroxide-reducing enzymes such as catalase, glutathione peroxidase, and peroxiredoxins, as well as higher endogenous levels of reactive oxygen species in cancer cells, leading to the less efficient degradation of H_2_O_2_ and increased susceptibility to P-AscH^−^ [31,32,43,46,47].

Following these findings, numerous clinical trials have examined the effects of P-AscH^−^ in combination with chemotherapy or chemoradiation therapy in the treatment of several cancer types. In 2012, Monti et al. published the results of their phase I clinical trial studying the effects of P-AscH^−^ when combined with gemcitabine and erlotinib in 14 subjects with metastatic pancreatic cancer [48]. They observed no significant increase in side effects or toxicity with P-AscH^−^ while also observing a decrease in tumor size in 8 of 9 patients. In 2013, Welsh et al. published similar findings from their phase I clinical trial observing the effects of P-AscH^−^ in combination with gemcitabine in metastatic and node-positive pancreatic cancer [49]. Instead of treating patients with a set dose of P-AscH^−^ (50 g, 75 g, or 100 g), as was done in Monti’s study, patients were treated based upon their plasma ascorbate levels following the previous day’s infusion. Welsh et al. demonstrated that plasma ascorbate concentrations of up to 30 mM could safely be achieved while also demonstrating a potential survival benefit, with mean progression-free survival (PFS) and mean overall survival (OS) of 26 weeks and 12 months, respectively, compared to a mean OS of 5–7 months for gemcitabine alone and 11 months for FOLFIRINOX at that time [7,50]. This was one of the first clinical trials that suggested that P-AscH^−^ may offer a survival benefit to cancer patients.

Additional phase I and II clinical trials in pancreatic, lung, glioblastoma, and ovarian cancer have demonstrated similar results. Ma et al. observed that patients who received P-AscH^−^ in combination with carboplatin and paclitaxel for stage III/IV ovarian cancer had significantly fewer grade 1 and 2 toxicities compared to patients who did not receive P-AscH^−^, with no increase in grade 3 and 4 toxicities [45]. This study again showed a potential survival benefit, with a prolongation of the median time for disease progression. In a phase I trial observing P-AscH^−^ effects in glioblastoma by Schoenfeld et al., patients receiving P-AscH^−^ in combination with temozolomide and radiation demonstrated mean PFS of 13.3 months and mean OS of 21.5 months, respectively, compared to 7 months and 14 months historically [33]. Multiple ongoing phase I and II clinical studies utilizing P-AscH^−^ in pancreatic, lung, breast, colorectal, bladder, prostate, glioblastoma, and myeloid malignancies will report their findings in the coming years. Recently published studies in pancreatic cancer, including an additional phase I clinical trial, have focused on the effects of P-AscH^−^ when given in combination with radiation therapy.

### 2.2. Pharmacologic Ascorbate Increases Radiation Toxicity in Pancreatic Cancer While Protecting Normal Tissue

While P-AscH^−^ has shown efficacy when combined with standard-of-care chemotherapies in both in vivo experiments and clinical trials, it has also shown promise as a radiomodulator [41,44,51,52,53,54,55]. Du et al. demonstrated that the addition of P-AscH^−^ to radiation significantly increased DNA damage and significantly decreased the clonogenic survival of multiple pancreatic cancer cell lines compared to radiation alone [52]. They also showed significantly decreased tumor volume and increased survival compared to radiation or P-AscH^−^ alone in a xenograft model. Furthermore, mice that received radiation alone demonstrated a significant reduction in jejunal crypt cells that was partially reversed with the addition of P-AscH^−^ treatment. These results suggested that P-AscH^−^ may not only be an effective adjuvant to chemoradiotherapy but may also offer radioprotection to normal cells. Alexander et al. further demonstrated that P-AscH^−^ significantly improves clonogenic survival, decreases DNA damage, and decreases collagen deposition in normal intestinal cells following radiation [53]. Normal tissue toxicity secondary to radiation damage can have devastating effects on patients. Complication rates are 50% when the nearby intestine receives a total of 60 Gy, making radiation therapy a relatively risky endeavor for pancreatic cancer patients [56]. In addition to its radiosensitization effects on cancer, P-AscH^−^ may also offer normal tissue protection to ensure that more patients can tolerate full, uninterrupted radiation regimens.

### 2.3. Long-Term Survival following P-AscH^−^ in Pancreatic Cancer

**Figure 1:**
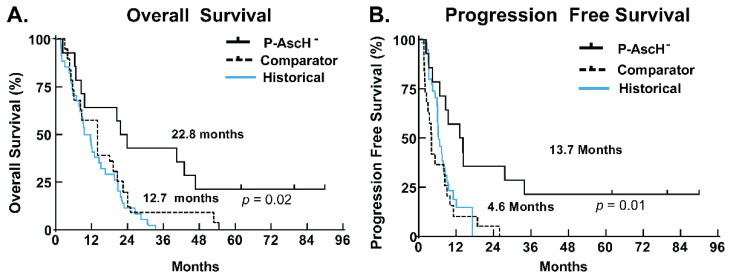
Survival analysis from phase I trial (NCT01852890). (**A**) Kaplan–Meier curve estimating median overall survival in subjects treated with P-AscH^–^ plus gemcitabine and radiation therapy as of 25 January 2022 (*n* = 14) was 22.8 months vs. 12.7 months in institutional controls treated with gemcitabine and radiation therapy (*n* = 19, Log-Rank test *p* = 0.02); (**B**) Kaplan–Meier curve demonstrating median progression-free survival in subjects treated with P-AscH^–^ plus gemcitabine and radiation therapy as of 25 January 2022 (*n* = 14) was 13.7 months vs. 4.6 months in institutional controls treated with gemcitabine and radiation therapy (*n* = 19, Log-Rank test *p* = 0.01). These data are updated from data previously published by Du et al. [60].

P-AscH^−^ has experienced a rebirth in the field of cancer therapy, with clinical trials underway in a variety of cancers [49,57]. The first phase I clinical trial to actively infuse P-AscH^−^ during the “beam on” time of radiation for the treatment of locally advanced pancreatic cancer, “Gemcitabine, Ascorbate, Radiation therapy for pancreatic cancer, phase I” (NCT01852890), was performed at the University of Iowa [53]. Pharmacologic ascorbate was administered concurrently with gemcitabine and radiation, where gemcitabine was administered weekly for 6 weeks and P-AscH^−^ was administered at 50–100 g during each radiotherapy treatment, either 28 fractions at 50.4 Gy or 25 fractions at 50 Gy. Fourteen subjects completed the protocol therapy between 2014 and 2017. Three study participants are now more than 5 years out from the completion of their neoadjuvant therapies as of January 2022, with the longest surviving participant out nearly 8 years (90 months), resulting in significant increases in both median overall survival (Figure 1A) and median progression-free survival (Figure 1B). Median overall survival for the P-AscH^−^/gemcitabine/radiation group is 22.8 months, with three long-term survivors (more than 5 years), compared to 12.7 months in the control group and 14 months historically for locally advanced pancreatic cancer [58]. Similarly, the median progression-free survival of 13.7 months in the P-AscH^−^ treatment group is also significantly longer than 4.6 months in the control group and roughly 3.8 months historically for all stages of pancreatic cancer [59]. Three long-term survivors from the relatively small sample size of a phase I trial suggests the efficacy of P-AscH^−^ as a chemotherapeutic agent and radiosensitizer. Currently, there are dozens of clinical trials using P-AscH^−^ in a variety of cancers, including phase II trials in pancreatic cancer utilizing P-AscH^−^ in combination with standard-of-care gemcitabine and nab-paclitaxel (NCT02905578) as well as more experimental regimens (NCT01905150).

## 3. Pathways for Hydrogen Peroxide Removal

Cancer cells’ decreased ability to neutralize the P-AscH^−^-induced increase in H_2_O_2_ has been shown to be secondary to the decreased expression and activity of enzymes responsible for removing H_2_O_2_, including catalase, glutathione peroxidase (GPx), and peroxiredoxins (Prx) [46,47,61,62]. Catalase, predominantly located in peroxisomes, has been shown to be the principal enzyme in H_2_O_2_ removal, especially in the presence of high amounts of H_2_O_2_, where it is responsible for removing as much as 99% of H_2_O_2_ in erythrocytes [63,64]. GPx and peroxiredoxin are considered more important for the removal of lower levels of H_2_O_2_ due to their higher affinity [64]. The catalytic activity of GPx is dependent on a regenerating system in which NADPH is used as an electron donor [65]. Utilizing two glutathione (GSH) molecules, GPx generates H_2_O and glutathione disulfide (GSSG) that is reduced back to glutathione (GSH) by glutathione reductase (GR) using NADPH. Any process that interferes with GPx activity, GR activity, GSSG recycling, or NADPH recycling can impair GPx turnover and reduce its effectiveness [66].

Another means by which to reduce H_2_O_2_-scavenging capacity is the targeting of the thioredoxin (Trx) redox buffer system. The thioredoxin system consists of thioredoxin and peroxiredoxin. Thioredoxin is a 12 kDa oxidoreductase necessary for a myriad of cellular processes, including deoxynucleoside triphosphate (dNTP) synthesis, transcriptional regulation, and antioxidant defense [67,68]. The disulfide exchange activity of thioredoxin is integral to the maintenance of peroxiredoxin. Peroxiredoxins are important H_2_O_2_ scavengers, with a reaction rate approaching diffusion-controlled limits (10^7–8^ M^−1^ s^−1^) and comparable to catalase (10^7^ M^−1^ s^−1^) and GPx (10^8^ M^−1^ s^−1^) [64,69,70,71]. Thus, the presence of sufficient reduced thioredoxin (Trx_(SH)2_) to maintain peroxiredoxin in the active (reduced) form is vital for cellular defense against H_2_O_2_. Following the two-electron reduction of a peroxiredoxin by reduced thioredoxin, thioredoxin disulfide (Trx_S-S_) is recycled back to reduced thioredoxin by thioredoxin reductase (TrxR), a flavin-containing selenoprotein, using reducing equivalents from NADPH [72,73]. Previous studies have shown that inhibition of the thioredoxin/thioredoxin reductase complex reduces peroxiredoxin function [2].

Targeting these pathways offers another potential avenue for cancer treatment. By decreasing cancer cells’ ability to degrade H_2_O_2_, oxidative stress within cancer cells may be further amplified, ultimately leading to enhanced cancer-specific cytotoxicity. A recent study examining the role of catalase in pancreatic cancer demonstrated that catalase knockout cells exhibited greater radiosensitization to P-AscH^−^ and that cancer cells in long-term survivors may express lower levels of catalase than cancer cells in short-term survivors [60]. These results suggest that inhibiting a portion of the peroxide removal system—catalase, glutathione reductase, thioredoxin reductase, or peroxiredoxin—may enhance the effectiveness of treatments aimed at increasing oxidative stress within cancer cells.

## 4. Auranofin

Auranofin (Au) is a gold-triethylphosphine compound originally FDA-approved in 1985 for the treatment of rheumatoid arthritis [74,75]. Au is one of three disease-modifying antirheumatic drugs (DMARDs) that contain gold, though it is the only drug in this group that can be administered orally due to its lipophilic properties [74]. Au is believed to exert multiple anti-inflammatory effects by altering the production and secretion of pro-inflammatory cytokines and modulating intracellular signaling pathways such as activating mitogen-activated protein kinases (MAPK), all of which play a role in rheumatoid arthritis disease severity and progression [76,77,78,79,80,81]. The exact mechanisms behind these effects are not completely understood but do seem to involve enzyme inhibition and appear to be dependent on the gold atom situated between a sulfur atom and the triethylphopshine group [82,83,84,85]. Additionally, Au has shown effectiveness outside of its immune-modulating effects and has garnered interest for its antibacterial, antiviral, cytoprotective, and anticancer potential [86,87,88,89,90,91].

### 4.1. Auranofin Use in Cancer

**Figure 2:**
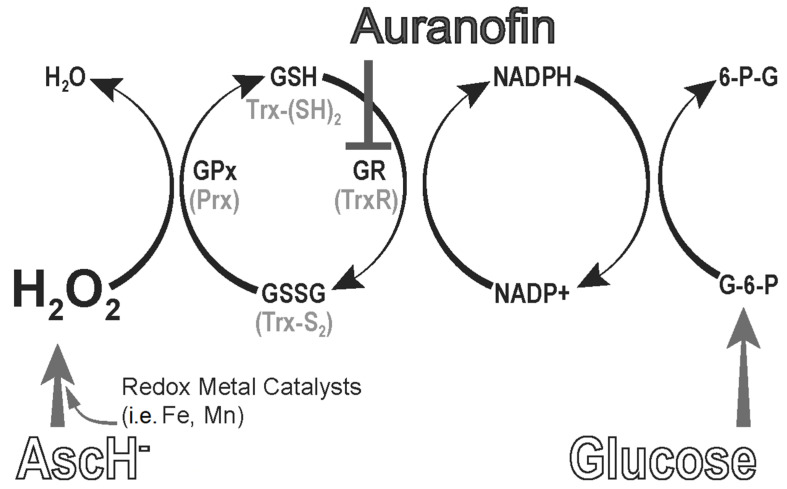
Thioredoxin and glutathione peroxidase (GPx) antioxidant enzyme systems and inhibitors. GSH = glutathione; GSSG = glutathione disulfide; GR = glutathione disulfide reductase; GPx = glutathione peroxidase; Trx-(SH)_2_ = reduced thioredoxin; Trx-S_2_ = oxidized thioredoxin; TrxR = thioredoxin reductase; Prx = peroxiredoxin; G-6-P = glucose-6-phosphate; 6-P-G = 6-phosphoglucono-δ-lactone. Inhibitors of the pathway are: Auranofin [103].

Over the past few decades, significant interest in the anticancer effects of Au has arisen. The key mechanism of action thought to be responsible for the anticancer effects of Au is thioredoxin reductase inhibition (Figure 2) [92,93]. Au serves as an electrophile and reacts with the selenocysteine residue in the active site of thioredoxin reductase by forming a covalent adduct and inactivating the enzyme [73]. Inhibition of thioredoxin reductase inhibits the flow of electrons from NADPH to peroxiredoxin by impairing the recycling of thioredoxin, leading to a subsequent reduction in peroxiredoxin activity and an increase in H_2_O_2_ [94]. Increasing H_2_O_2_ via this mechanism may lead to additional cancer-specific cytotoxicity, as discussed previously [31,95]. Several in vitro studies have demonstrated a synergistic induction of apoptosis when Au is given in combination with various chemotherapy agents in breast cancer cell models [96,97,98]. In one study, Lee et al. demonstrated that the combination of Au and mesupron, a urokinase-type plasminogen activator inhibitor currently undergoing clinical trials in breast cancer, promoted the inhibition of breast cancer cell proliferation and induced cell cycle arrest and apoptosis, while also significantly increasing reactive oxygen species within cancer cells [97]. Similar studies in colon, ovarian, and lung cancer cell lines demonstrating synergistic increases in cancer cell death show that Au may be an effective anticancer agent when combined with other chemotherapy agents [99,100,101]. Based on these findings, a previous study hypothesized that Au may sensitize cancer cells to P-AscH^−^ by impairing hydroperoxide removal [102]. To test this, pancreatic ductal adenocarcinoma cancer cells were treated with inhibitors of hydroperoxide metabolism. Data show that targeting of the thioredoxin system with Au inhibits thioredoxin reductase activity and sensitizes pancreatic cancer cells to P-AscH^−^-generated H_2_O_2_. The combination of Au and P-AscH^−^ also significantly increases the sensitivity of pancreatic cancer cells to ionizing radiation. Based on these results, repurposing Au in combination with P-AscH^−^ as a chemotherapy and radiomodulatory regimen may provide a potentially promising and translatable new treatment for pancreatic cancer. For all experiments, Au was dissolved in PBS containing 1% EtOH.

### 4.2. Au Inhibits Thioredoxin Reductase Activity in Pancreatic Cancer Cells

**Figure 3:**
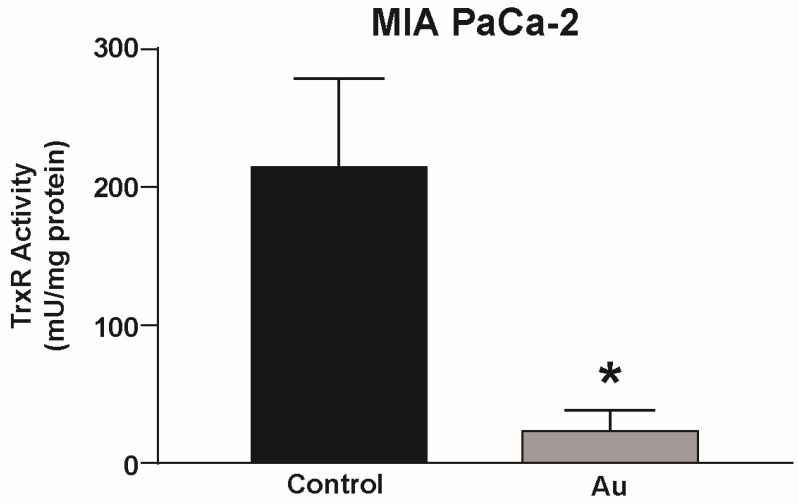
Au inhibits thioredoxin reductase activity in pancreatic cancer cells. MIA PaCa-2 cells were incubated with 1 µM Au for 3 h prior to measuring thioredoxin reductase activity. Au significantly reduced thioredoxin reductase activity compared to control. Data represent thioredoxin reductase activity in mU/mg protein ± SE (* *p* < 0.05; two-tailed unpaired Student’s *t* test). Reprinted/adapted with permission from [102,103]. Copyright 2017 Elsevier.

To determine if Au inhibits thioredoxin reductase activity in pancreatic cancer cells, MIA PaCa-2 pancreatic cancer cells were treated with Au for 3 h and thioredoxin reductase activity was measured by spectrophotometrically following the reduction of 5,5-dithio-bis-(2-nitrobenzoic acid) (DTNB) utilizing a thioredoxin reductase assay kit. Activity was normalized to the protein concentration determined by the Lowry protein assay. Au was shown to significantly decrease thioredoxin reductase activity in pancreatic cancer cells (Figure 3) [102].

### 4.3. Auranofin Sensitizes Pancreatic Cancer Cells to P-AscH^−^

**Figure 4:**
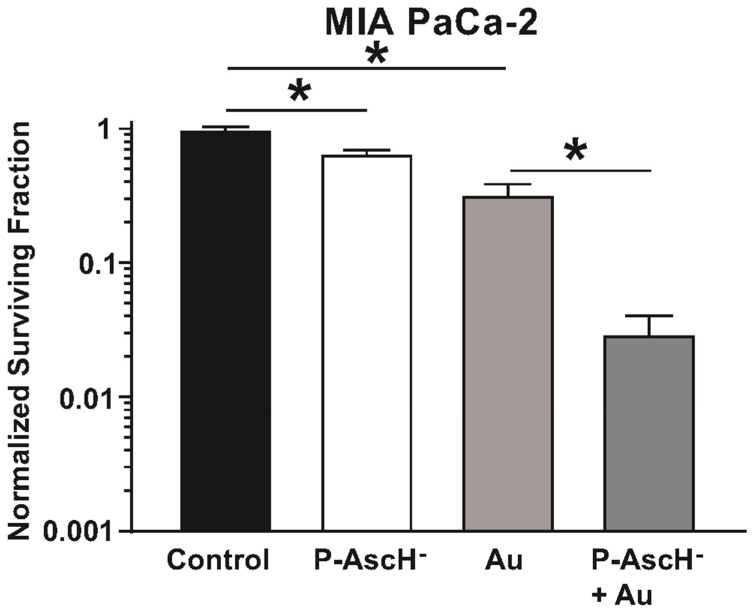
Au sensitizes pancreatic cancer cells to P-AscH^−^. Clonogenic cell survival of MIA PaCa-2 cells incubated with Au (1 µM for 3 h) followed by 2 mM P-AscH^−^ (1 h) was significantly decreased compared to control and to either treatment alone. Treatment with either Au or P-AscH^−^ also decreased clonogenic survival compared to control. Data represent normalized surviving fractions compared to control ± SE (* *p* < 0.01; one-way ANOVA with Tukey’s multiple comparisons). Reprinted/adapted with permission from [102,103]. Copyright 2017 Elsevier.

As described above, P-AscH^−^ is selectively cytotoxic to cancer cells due to the generation of high amounts of H_2_O_2_ [24,25,31]. Furthermore, the cytotoxic effects of P-AscH^−^ are enhanced in catalase knockout cell lines [60]. Based on these findings, it was hypothesized that inhibition of thioredoxin reductase by Au would disrupt the thioredoxin-dependent hydroperoxide scavenging system and sensitize pancreatic cancer cells to P-AscH^−^ treatment [102]. To test this, exponentially growing MIA PaCa-2 cells were treated with Au for 3 h prior to P-AscH^−^ treatment for 1 h. Stock solutions of L-ascorbic acid were prepared under argon and stored in screw-cap glass vials at 4 °C. The utilized dose of P-AscH^−^ has been previously demonstrated to result in 50% clonogenic survival (ED_50_) of the MIA PaCa-2 cell line over many iterations of clonogenic survival analysis [43]. Following treatment, cells were detached, counted, and plated at designated densities, and allowed to grow for 10–14 days. Surviving fractions were calculated and normalized to control plates. Auranofin alone decreased clonogenic survival compared to the control, while the combination treatment of P-AscH^−^ with Au further decreased clonogenic survival compared to either treatment alone (Figure 4) [102].

**Figure 5:**
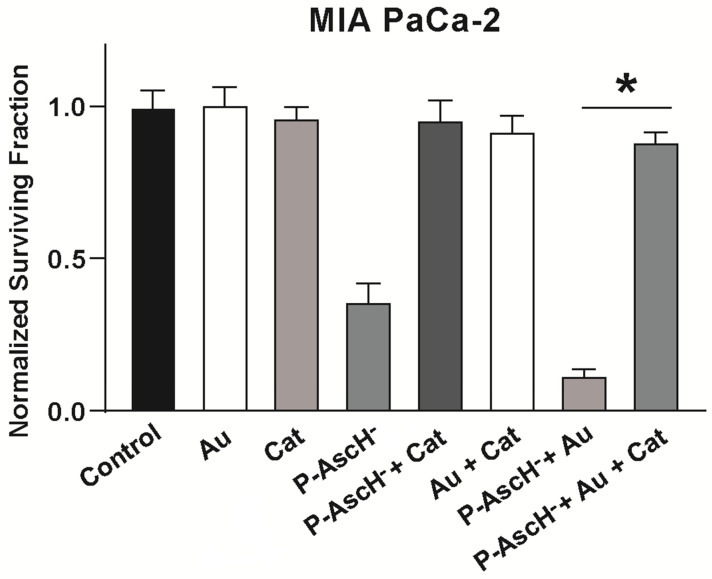
Catalase reverses killing induced by the combination of Au and P-AscH^−^. MIA PaCa-2 cells were evaluated for clonogenic cell survival after treatment with either catalase (Cat, 100 U/mL, 1 h), Au (500 nM, 24 h), and/or P-AscH^−^ (1 mM, 1 h). The combination of P-AscH^−^ and Au significantly decreased clonogenic cell survival compared to P-AscH^−^, and the addition of catalase completely reversed the decrease. Data represent normalized surviving fractions compared to controls ± SE (* *p* < 0.05; one-way ANOVA with Tukey’s multiple comparisons). Reprinted/adapted with permission from [102,103]. Copyright 2017 Elsevier.

Cancer cell killing by P-AscH^−^ has been shown to be dependent on extracellular H_2_O_2_ [33,41]. Thus, the overexpression of catalase or addition of bovine catalase in media was hypothesized to partially or completely rescue cancer cells from toxicity, mediated by the combination of P-AscH^−^ and Au. P-AscH^−^ was again dosed at the ED_50_ of the cell line, as determined in previous experimentation [43]. P-AscH^−^ caused slightly more cell death in this set of experiments (~40% vs. ~60% in Figure 4), though this small difference is not uncommon [43]. The addition of bovine catalase to cell media prevented the synergistic effect of Au/P-AscH^−^ in pancreatic cancer cells (Figure 5) [102]. These findings support the hypothesis that H_2_O_2_ is responsible for the effects of P-AscH^−^ when cells are treated with Au and P-AscH^−^ in combination.

### 4.4. Au Combined with P-AscH^−^ Sensitizes Pancreatic Cancer Cells to Ionizing Radiation

**Figure 6:**
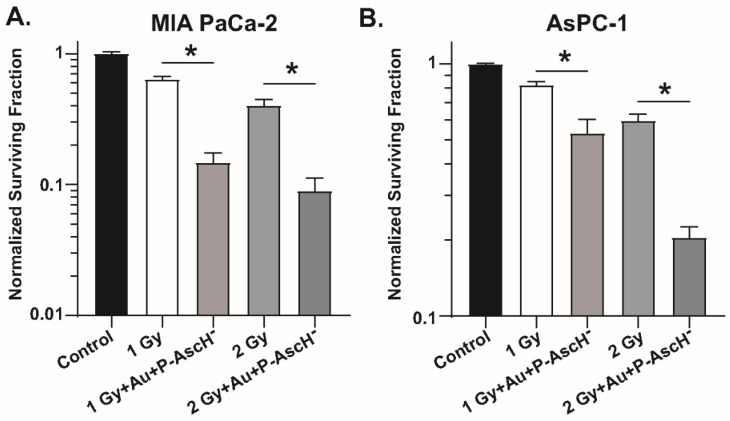
Au + P-AscH^−^ radiosensitizes pancreatic cancer cell lines. (**A**) MIA PaCa-2 and (**B**) AsPC-1 cells were evaluated for clonogenic survival after treatment with either irradiation alone (1–2 Gy) or in combination with Au (500 nM, 24 h), P-AscH^−^ (1–2 mM, 1 h), and irradiation (1–2 Gy). The combination of Auranofin and P-AscH^−^ significantly reduced clonogenic cell survival of MIA PaCa-2 and AsPC-1 cells at both 1 and 2 Gy. Data represent normalized surviving fractions compared to controls ± SE (* *p* < 0.05; two-tailed unpaired Student’s *t* test). Reprinted/adapted with permission from [102,103]. Copyright 2017 Elsevier.

P-AscH^−^ has been shown to sensitize cancer cells to ionizing radiation in an H_2_O_2_-dependent manner in both in vitro and in vivo studies and has shown promise in recent phase I clinical trials [52,53]. To determine the radiosensitizing effects of Au and P-AscH^−^ in combination in pancreatic cancer cell lines, exponentially growing MIA PaCa-2 and AsPC-1 pancreatic cancer cells were radiated with 1–2 Gy ionizing radiation with or without treatment with Au for 24 h prior to treatment with P-AscH^−^ for 1 h. Cells were then detached, counted, and plated at designated densities and allowed to grow for 10–14 days and surviving fractions calculated as described previously [43]. The combination of Au and P-AscH^−^ caused significant cell death (~90%) when combined with radiation (Figure 6) [102]. These data suggest that combining Au with P-AscH^−^ has the possibility of increasing radiation-induced toxicity.

### 4.5. Au and P-AscH^−^ Potential in Cancer Therapy

These studies demonstrate that inhibition of the thioredoxin antioxidant system by the FDA-approved anti-rheumatic agent Au inhibits thioredoxin reductase activity and sensitizes pancreatic cancer cell lines to treatment with P-AscH^−^ in a manner that is dependent on H_2_O_2_. In addition, the combination of Au and P-AscH^−^ sensitizes pancreatic cancer cells to radiation therapy. The selective sensitivity imposed by thioredoxin reductase inhibition may be due to the impairment of H_2_O_2_ metabolism by peroxiredoxin, resulting in enhanced H_2_O_2_-mediated oxidative damage, leading to cell death from oxidative protein, lipid, and/or DNA damage. Alternatively, the unique role of the thioredoxin system in redox signaling may also explain the efficacy of Au. Due to the environment around the redox-active cysteines, peroxiredoxins are uniquely amenable to oxidation by H_2_O_2_ [104]. In fact, peroxiredoxins exhibit a reactivity toward H_2_O_2_ approximately six to eight orders of magnitude higher than other redox-regulated proteins [105]. This sensitivity allows peroxiredoxin to outcompete other thiols for H_2_O_2_ and serve as a medium through which oxidative equivalents can be transduced as a signal [106,107]. Indeed, peroxiredoxins exhibit a significant influence over cell death signaling [108,109,110] through the oxidative modification of several targets, such as p38 [111], ERK [109], ASK1 [112,113], Akt [111,114], and STAT3 [115], among many others.

Studies have shown that peroxiredoxin hyper-oxidation sensitizes cells to cell death signaling induced by agents that produce H_2_O_2_ [111]. Other groups have shown that Auranofin-induced peroxiredoxin oxidation sensitizes triple-negative breast cancer and malignant B-cells to P-AscH^−^ [116,117]. Peroxiredoxin hyper-oxidation induced by the combination of a decreased capacity to recycle reduced thioredoxin and enhanced H_2_O_2_ generated by P-AscH^−^ may cause a synergistic enhancement of cell death signaling. Thus, the combination of Au and P-AscH^−^ may serve as a highly effective means to exploit the signaling of the peroxiredoxin system to induce tumor cell-specific cell death. Future experiments can interrogate the role of peroxiredoxin signaling in cell death induced by Au/Asc by using cell lines depleted of select peroxiredoxins [106].

## 5. Conclusions

Pancreatic cancer continues to carry an extremely poor prognosis and remains the third-leading cause of cancer deaths, despite advances in chemotherapy and radiation protocols. Pharmacologic ascorbate has shown promise as an effective adjunct therapy, with phase I and phase II clinical trials suggesting a survival benefit compared to standard-of-care therapies. Ongoing clinical trials in multiple cancer types will help to further elucidate the efficacy and role of pharmacologic ascorbate in cancer therapy. The effects of P-AscH^−^ on normal tissue have yet to be delineated. However, recent studies demonstrate radioprotective effects. This aspect of P-AscH^−^ therapy could improve quality of life for patients and help patients tolerate higher doses of chemotherapy and/or radiation, providing additional incentive for its use. In line with previous studies’ observations in other cancers, Au may also be useful as an adjunct therapy in the treatment of pancreatic cancer. Previous supporting data demonstrate that Au inhibits thioredoxin reductase and sensitizes pancreatic cancer cells to P-AscH^−^ in a manner that is dependent on H_2_O_2_. The combination of these two clinically available agents and their roles as radiomodulators presents an exciting avenue to enhance tumor responses to chemoradiation therapies and should be explored in future experimentation by comparing Auranofin to other current standard-of-care regimens. Additionally, studying the Auranofin mechanism in cancer treatment could offer significant insight into other models and techniques for exploiting H_2_O_2_-induced cytotoxicity in cancer.
